# L‐F001, a multifunctional fasudil–lipoic acid dimer, antagonizes hypoxic–ischemic brain damage by inhibiting the TLR4/MyD88 signaling pathway

**DOI:** 10.1002/brb3.3280

**Published:** 2023-10-11

**Authors:** Ruiyu Zhou, Liqiang Wu, Ni Jin, Sha Sha, Ying Ouyang

**Affiliations:** ^1^ Sun Yat‐sen Memorial Hospital Sun Yat‐sen University Guangzhou China; ^2^ The Affiliated Kashi Hospital Sun Yat‐sen University Kashi China; ^3^ Guangdong Provincial Emergency Hospital Guangzhou China

**Keywords:** anti‐inflammation, hypoxic–ischemic brain damage, L‐F001, prognosis, TLR4/MyD88

## Abstract

**Introduction:**

Neonatal hypoxic–ischemic brain damage (HIBD) is a serious inflammatory injury. At present, the standard treatment for this disease is hypothermia therapy, and the effect of drug intervention is still limited. L‐F001 is a compound of fasudil and lipoic acid. Previous in vitro experiments have confirmed that L‐F001 has anti‐inflammatory neuroprotective functions. However, its therapeutic effect on neonates with HIBD remains unknown. This study was aimed at exploring the therapeutic effect of L‐F001 on HIBD rats.

**Methods:**

The newborn rats were divided into three groups: Sham operation group, HIBD group, and HIBD + L‐F001 group. HE staining, Nissil staining, the immunofluorescence of iNOS and COX‐2, ELISA (IL‐1β, IL‐6, TNF‐α, and IL‐10), and western blotting analyses were performed to determine the therapeutic effect of L‐F001. Finally, we evaluated the growth and development of each group by measuring body weight.

**Results:**

The hippocampal structure of HIBD rats was disordered, and the Nissil body was small and shallow. The expressions of iNOS and COX‐2 in HIBD rats were increased, whereas the expressions of IL‐1β, IL‐6, and TNF‐α in plasma were upregulated, and the expression of IL‐10 was decreased. L‐F001 could improve the tissue structure and reduce the expression of iNOS and COX‐2 in HIBD rats. Meanwhile, L‐F001 could also reduce the expression of pro‐inflammatory cytokines and restore the content of anti‐inflammatory cytokines in plasma. We further found that the TLR4 pathway was activated after hypoxic‐ischemia in neonatal rats. L‐F001 could inhibit the activation of TLR4 pathway. Finally, we found that after L‐F001 treatment, the body weight of HIBD rats increased significantly compared with the untreated group.

**Conclusions:**

L‐F001 antagonizes the inflammatory response after hypoxic‐ischemia by inhibiting the activation of the TLR4 signaling pathway, thus playing a neuroprotective role. L‐F001 may be a potential therapeutic agent for neonatal HIBD.

## Graphical abstract

L‐F001 is a compound of fasudil and lipoic acid. L‐F001 antagonizes the inflammatory response after hypoxic‐ischemia by inhibiting the activation of the TLR4 signaling pathway, thus playing a neuroprotective role. L‐F001 may be a potential therapeutic agent for neonatal HIBD.

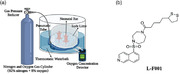

## 1 INTRODUCTION

Neonatal hypoxic–ischemic brain damage (HIBD) is one of the leading causes of neonatal death, accounting for one in five neonatal deaths worldwide (Novak et al., 2018). In addition, HIBD can lead to varying degrees of neurological damage, among which the serious consequences are cerebral palsy and mental retardation (Kurinczuk et al., 2010). The pathophysiology of HIBD is very complex. After hypoxic‐ischemia, cells undergo stages of damage and recovery with a continuous feedback loop. In the absence of oxygen, the excitatory amino acid, glutamate, accumulates inside the cell. Glutamate allows increased Ca^2+^ flow into cells, activates lipase and NO synthases, disrupts mitochondrial structure and function, and triggers the release of oxygen radicals. Oxygen radicals cause cell death not only due to direct hypoxic–ischemic damage but also due to ischemia reperfusion (reoxygenation), which promotes the continuous occurrence of necrosis–apoptosis (Hassell et al., 2015; Kriz, 2006). Dead cells in turn induce inflammation, which further releases oxygen radicals. Besides, newborn brains lack antioxidant systems, making them vulnerable to hypoxic–ischemic damage (Forman et al., 2010; Robles et al., 2001). Therefore, preventing inflammation and oxygen radical damage is very important for neonates with HIBD.

The current standard treatment for neonates with HIBD is to start hypothermia within 6 h after birth (Silveira & Procianoy, 2015). Despite the benefits of hypothermia therapy, nearly half of the babies treated still died or were disabled. To prevent death or disability in one child with HIBD, we have to treat seven to eight children with hypothermia (Bonifacio & Hutson, 2021; Glass, 2018). At present, there is still no drug intervention that has been proven to be useful. So the focus eventually shifted to drugs, such as oxygen radical scavengers, calcium channel blockers, and excitatory amino acid antagonists. In any case, we need a new drug that can enhance the effects of hypothermia to further improve outcomes. In the view of the complex pathogenesis of HIBD, the therapeutic strategy of single drug and single target is not effective. In order to increase the efficacy and reduce the side effects caused by the combination of drugs, we synthesized a novel fasudil–lipoic acid dimer L‐F001. Previous studies have shown that L‐F001 can significantly inhibit lipopolysaccharide‐induced neuroinflammation and reduce microglial activation in vitro by inhibiting nuclear factor‐κb (Nf‐κb) and activating the nuclear factor (erythroid‐derived 2)‐like 2 (Nrf‐2) pathway (Luo et al., 2017). It can be seen that L‐F001 has antioxidant and anti‐inflammatory neuroprotective effects. However, its therapeutic effect in neonatal rats with HIBD remains unclear.

Therefore, in order to investigate whether L‐F001 can improve brain damage and prognosis in neonatal HIBD rats, we conducted this study. By establishing sham operation group, HIBD group, and L‐F001 treatment group, we compared the brain structure and function changes of rats in each group, explored the therapeutic effect of L‐F001 on newborn HIBD rats, and evaluated whether it can improve the growth and development of HIBD rats.

## 2 MATERIALS AND METHODS

### 2.1 Materials

**FIGURE 1:**
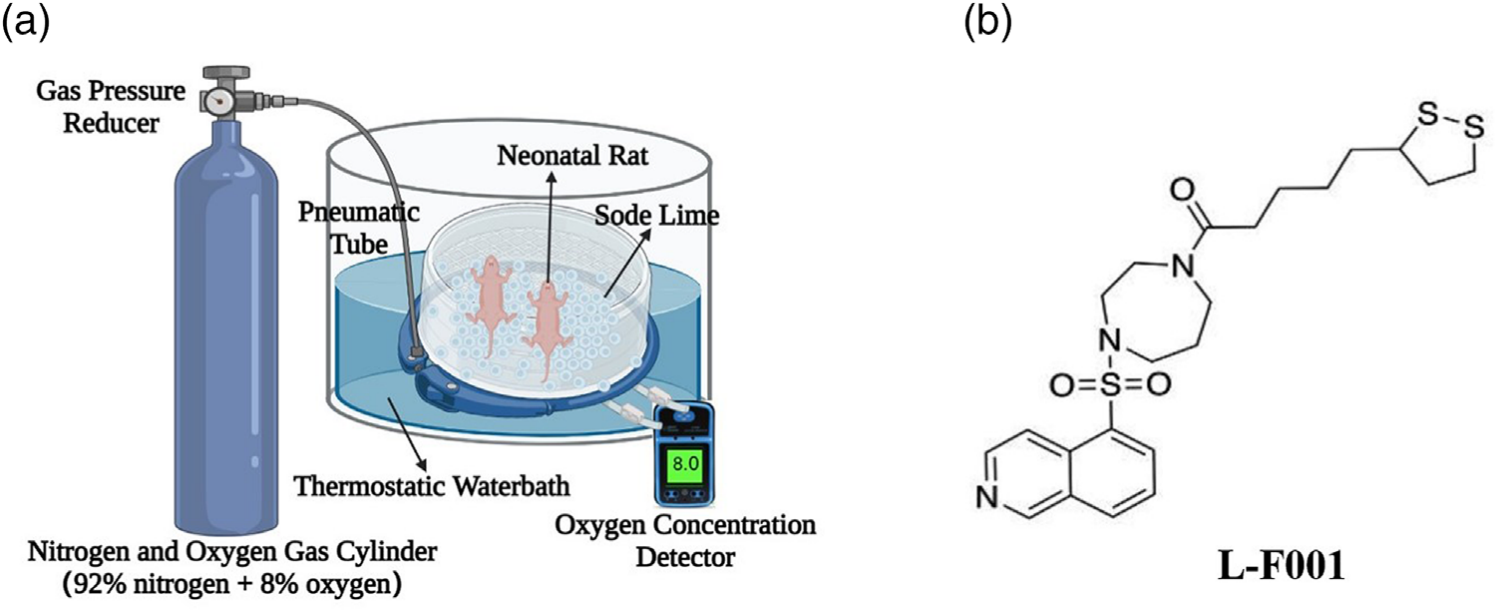
(a) Schematic diagram of hypoxic device and (b) structural formula of L‐F001.

Specific Pathogen Free (SPF) grade healthy 15‐day pregnant female Wistar rats were purchased from Vital River Co., Ltd. Oxygen concentration detector was purchased from Skesen. Nitrogen and oxygen gas cylinders were purchased from gas plant. Paraformaldehyde, chloral hydrate, sodium lime, and isoflurane were purchased from Macklin. The hypoxic device was made by ourselves (Figure 1a). ELISA kits were purchased from Elabscience. Primary antibodies against TLR4, MyD88, Nf‐κb p65, Nf‐κb pp65, and GAPDH were purchased from Proteintech. DAPI, hematoxylin‐eosin (HE), and Nissil dyeing solutions were purchased from Beyotime. L‐F001 (Figure 1b) (purity >98%) was previously synthesized, and its chemical characterization (mass spectrum, purity, etc.) can be seen in the previous report (Chen et al., 2014).

### 2.2 Methods

Wistar rats weighing 12–15 g at 6 days old were selected for the experiment. Experimental groups: sham operation group (sham group), HIBD group, and L‐F001 treatment group (HIBD + L‐F001 group). The rats were randomly assigned to the group. Both female and male newborn rats, the body weight of which met the standard could be included in the group. There were 20 rats in each group. This part of animal experiments has passed the examination of the Experimental Animal Welfare Ethics Management Committee of Sun Yat‐sen University (No: 2022000483).

### 2.3 Construction of rat models

In the sham group, we isolated the unilateral common carotid artery without ligation and then sutured the skin. In HIBD group, unilateral common carotid artery was separated and ligated with a double‐strand 6‐0 suture. Then the rats were put back to the mother mice to rest for 90 min and then placed in the hypoxic device for 2.5 h. The sham group and the HIBD group were given 20 μL intraperitoneal injection of normal saline 10 min after modeling, twice a day at an interval of 8 h for 3 days. In the treatment group, 20 μL L‐F001 (35 mg/kg) was intraperitoneally injected 10 min after modeling, twice a day at an interval of 8 h for 3 days. After 72 h of modeling, hippocampal brain tissue was extracted after cardiac perfusion, and paraffin embedding was performed.

### 2.4 HE stain

HE staining was performed on the hippocampus. Hematoxylin staining: The rewarmed sections were placed in a vessel containing hematoxylin, soaked for 3–5 min, and then the sections were turned blue with 0.6%–0.7% ammonia solution. Eosin staining: Different concentrations of alcohol were used for gradient dehydration (85%−95%) and then soaked in eosin staining solution for 5 min to stain the sections. When the staining was complete, we could see that the nucleus was stained blue and the cytoplasm was red.

### 2.5 Nissil stain

Nissil staining was performed on the hippocampus. Nissil staining: Brain tissue sections were put into the dye solution for 1–5 min, washed with water, differentiated with 1% glacial acetic acid, and finally baked dry. We added xylene into the sections for 5 min until the sections were transparent. Then we used neutral gum to seal the piece. Result interpretation: The brain tissue Nissil body was dark blue with light blue background.

### 2.6 Immunofluorescence

Immunofluorescence was performed on the hippocampus. The slices were immersed in a repair box containing EDTA antigenic repair buffer (pH 8.0) and baked in a microwave oven for antigenic repair. The primary antibody solution was added to the tissue and placed in a refrigerator at 4°C overnight. Then the secondary antibody solution was added and incubated at room temperature for 60 min. We used DAPI staining solution to restain the nuclei of tissues. The sections were observed and photographed using a fluorescence microscope (Olympus BX63). DAPI (blue): Ex 330–380 nm, Em 420 nm. CY3 (red): Ex 510–560 nm, Em 590 nm. FITC (green): Ex 465–495 nm, Em 515–555 nm. ImageJ was used to conduct quantitative statistics on the fluorescence intensity of DAPI and marker in the image, respectively. Then the relative fluorescence intensity of marker/DAPI was calculated, and finally, the comparison between groups was carried out. Each group has more than 200 cells.

### 2.7 ELISA

Cardiac blood was taken from rats and centrifuged at 3000 rpm/min for 20 min to separate plasma. Cytoinflammatory factors (IL‐1β, IL‐6, IL‐10, TNF‐α) in plasma were detected by ELISA kits. Each group had three multiple wells, and the independent experiment was repeated three times.

### 2.8 Western blotting

We extracted protein from the hippocampus and measured the protein content. An equal amount of protein (20 μg/lane) was electrophoretically isolated on SDS–PAGE and transferred to polyvinylidene difluoride (PVDF) membranes. PVDF membrane was sealed with protein‐free rapid sealing solution at room temperature for 30 min. The membranes were incubated overnight with specific primary antibodies at 4°C. The membrane was then incubated with the secondary antibody at room temperature for 60 min. We used the enhanced chemiluminescence detection system (Syngen G: BOX Chemi XT4) for protein detection.

### 2.9 Statistical analysis

Data from three to five independent experiments were expressed as mean ± SEM. The homogeneity of variance was tested by Levene. Western blotting was quantified by ImageJ. Univariate analysis of variance (ANOVA) was used to measure differences between groups. The difference between the two groups was examined by the Student *t* test. The repeated measurements between groups were compared using two‐way ANOVA and the Tukey test. *p* < .05 was considered statistically significant.

## 3 RESULTS

### 3.1 L‐F001 improved tissue and structure damage of hippocampus in HIBD rats

**FIGURE 2:**
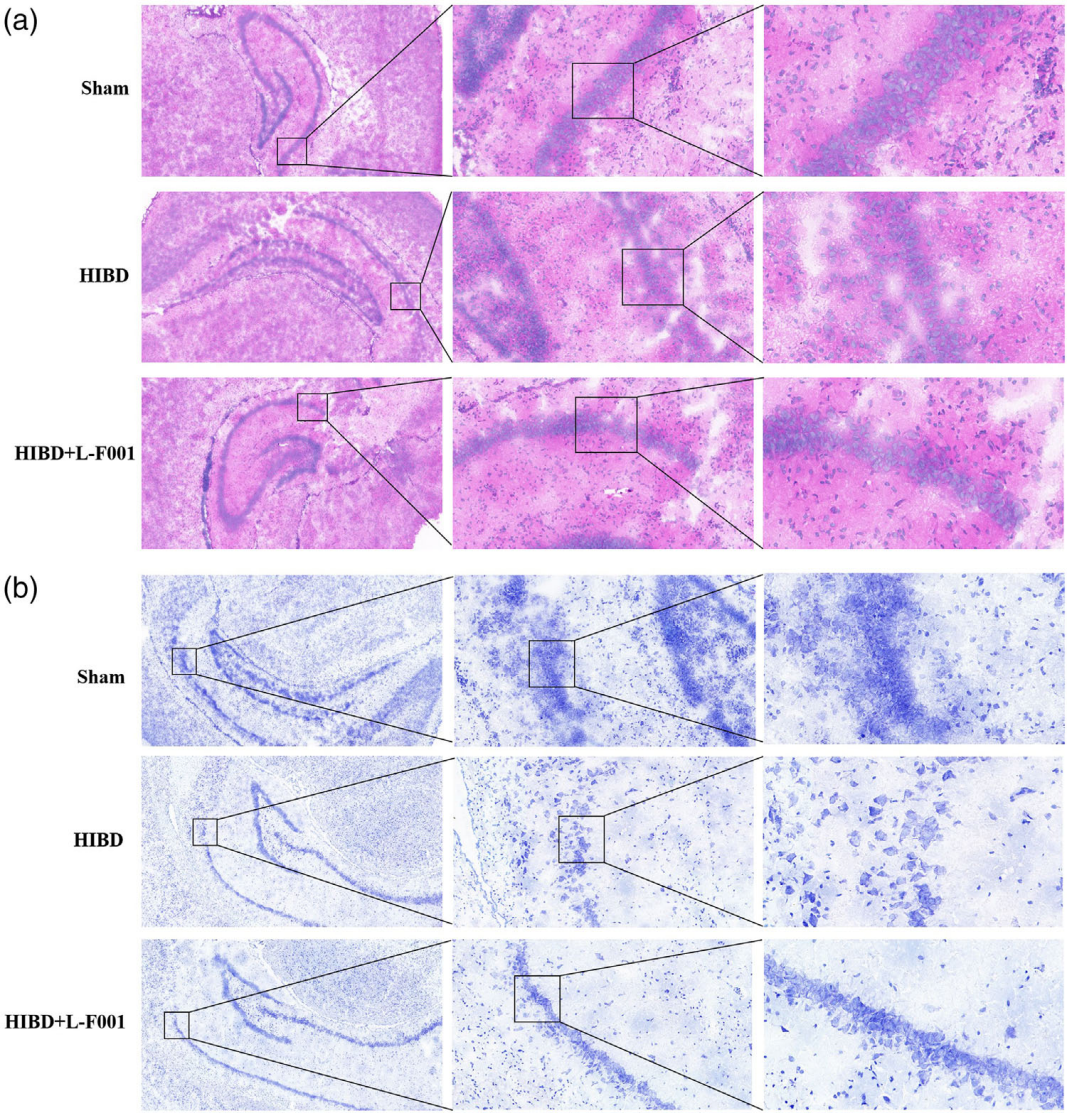
L‐F001 improved tissue and structure damage of hippocampus in hypoxic–ischemic brain damage (HIBD) rats: (a) HE staining of rat hippocampus; (b) Nissil staining of rat hippocampus. The scales from left to right are 200, 50, and 20 μm.

All the animals in the model group had abnormal changes of different degrees, including restlessness and tumbling 5–10 min after entering the hypoxic chamber and then gradually appeared muscle tremor, head tremor, inability to turn over, spontaneous or left rotation of the tail clamp, and even convulsions, which alternately appeared with consciousness disturbance. It can be seen that the model is successfully established. The cells in the hippocampal CA1 region of the sham group were normal in morphology and neatly distributed in multiple layers. Nuclear and cytoplasm staining was uniform and clear. Compared with sham group, the cells in HIBD group had different sizes, some of them were reduced in volume, arranged disordered, the original layers were destroyed, and the cell layers were reduced. Some cells showed uneven staining and nuclear color enhancement. After L‐F001 treatment, the cells in hippocampal CA1 region were more abundant than those in HIBD group, with more orderly arrangement and distribution. The cell morphology and size were more uniform than those in HIBD group (Figure 2a). The morphology of neurons in hippocampal CA1 region of rats in sham group was regular. The Nissil bodies were dark blue in color, large in size and abundant in number, neatly arranged, and richly layered. Compared with the sham group, the number of neurons in the hippocampal CA1 region of rats in the HIBD group was significantly reduced, and the morphology was irregular. The Nissil bodies were small and noticeably lighter in color. The neurons were disorganized and had no hierarchy. After L‐F001 treatment, the number of neurons increased compared with that in HIBD group, and the color of Nissil bodies became darker (Figure 2b). It is suggested that intraperitoneal injection of L‐F001 can improve the destruction of hippocampus structure and the decline of neuron function caused by HIBD.

### 3.2 L‐F001 decreased the expression of iNOS and COX‐2 in hippocampus of HIBD rats

**FIGURE 3:**
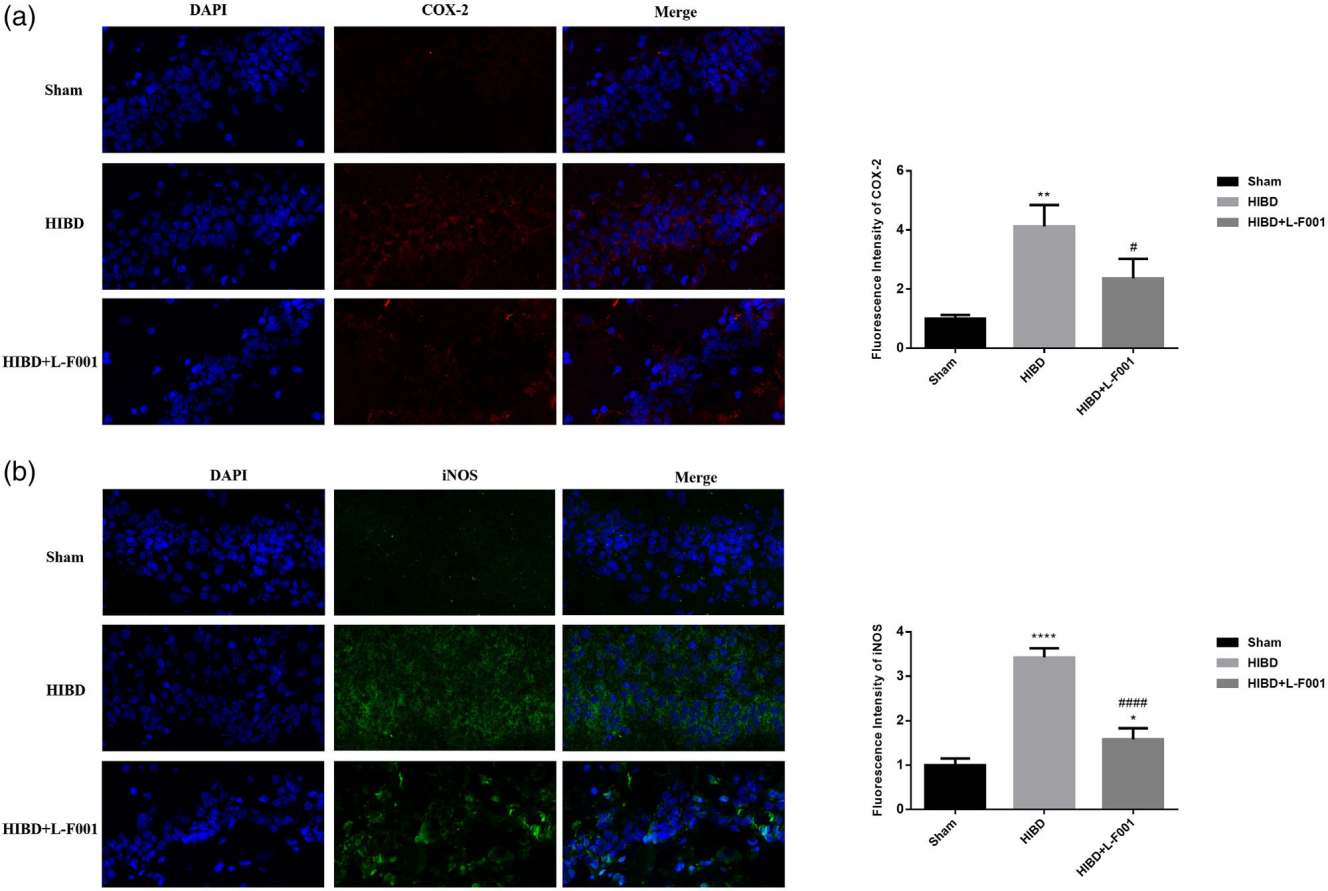
L‐F001 decreased the expression of iNOS and COX‐2 in hippocampus of hypoxic–ischemic brain damage (HIBD) rats: (a) immunofluorescence and statistical analysis of COX‐2. (b) Immunofluorescence and statistical analysis of iNOS. The scales from left to right are 200, 50, and 20 μm. Compared to the sham group: ^*^ *p* < .05, ^**^ *p* < .01, ^****^ *p* < .0001. Compared to the HIBD group: ^#^ *p* < .05, ^####^ *p* < .0001 (*n* = 6).

DAPI staining makes the nucleus blue. COX‐2 and iNOS fluoresce in red and green, respectively. iNOS and COX‐2 immunofluorescence were detected in the hippocampal tissues of each group. Compared with sham group, the iNOS and COX‐2 immunofluorescence intensities in HIBD group were significantly enhanced. After the intraperitoneal injection of L‐F001, the iNOS and COX‐2 fluorescence intensities in hippocampal tissue decreased significantly (Figure 3). It is suggested that L‐F001 can relieve the oxidative stress of hippocampus induced by HIBD after treatment.

### 3.3 L‐F001 decreased the expression of pro‐inflammatory cytokines IL‐1β, IL‐6, and TNF‐α and increased the secretion of anti‐inflammatory cytokines IL‐10 in plasma of HIBD rats

**FIGURE 4:**
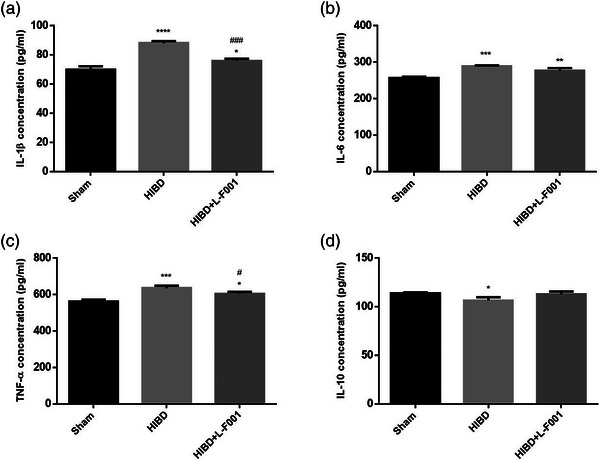
Changes of plasma inflammatory cytokines in rats: (a) IL‐1β; (b) IL‐6; (c) TNF‐α; (d) IL‐10. They were measured by ELISA kits. Compared to the sham group: ^*^ *p* < .05, ^**^ *p* < .01, ^***^ *p* < .001, ^****^ *p* < .0001. Compared to the hypoxic ischemic brain damage (HIBD) group: ^#^ *p* < .05, ^###^ *p* < .001 (*n* = 6).

We used ELISA kits to detect the content of inflammatory cytokines in rat plasma. Compared with sham group, the expression levels of pro‐inflammatory cytokines IL‐1β, IL‐6, and TNF‐α were significantly increased in HIBD group, whereas the expression levels of anti‐inflammatory cytokines IL‐10 were significantly decreased. After the intraperitoneal injection of L‐F001, the plasma pro‐inflammatory cytokine expression level of rats was significantly decreased compared with that of HIBD group, whereas the anti‐inflammatory cytokine expression level was slightly increased (Figure 4). It is suggested that the intraperitoneal injection of L‐F001 can improve the inflammatory response caused by HIBD.

### 3.4 L‐F001 inhibited the activation of the TLR4 signaling pathway

**FIGURE 5:**
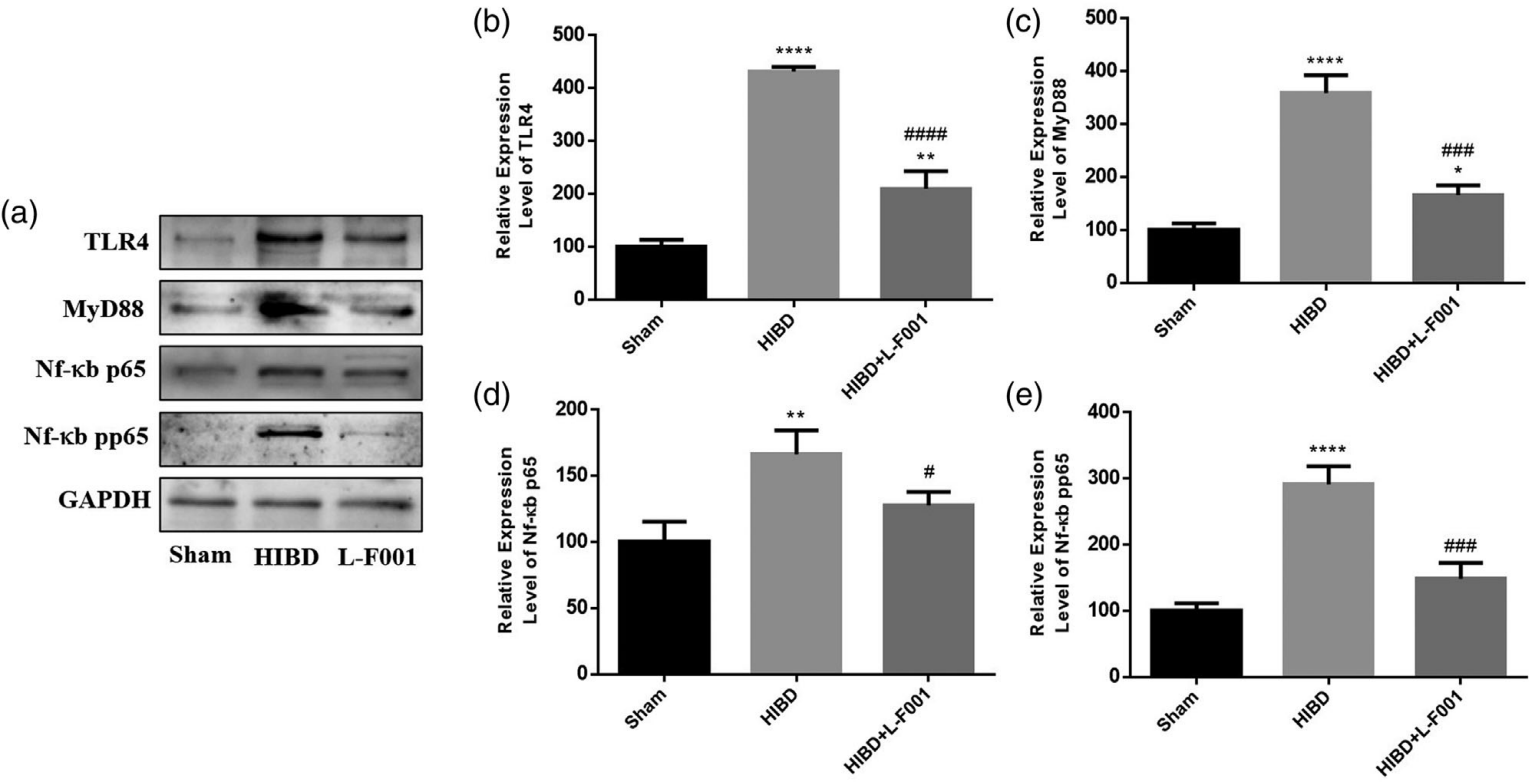
L‐F001 inhibited the activation of the TLR4 signaling pathway: (a) TLR4 pathway–associated protein bands; (b) statistical analysis of TLR4; (c) statistical analysis of MyD88; (d) statistical analysis of nuclear factor‐κb (Nf‐κb) p65; (e) statistical analysis of Nf‐κb pp65. Compared to the sham group: ^*^ *p* < .05, ^**^ *p* < .01, ^****^ *p* < .0001. Compared to the hypoxic ischemic brain damage (HIBD) group: ^#^ *p* < .05, ^###^ *p* < .001, ^####^ *p* < .0001 (*n* = 6).

We then used western blotting to further explore the potential molecular mechanism of L‐F001 influencing the inflammatory response. The results showed that the expression levels of TLR4, MyD88, and Nf‐κb p65 and pp65 proteins in the hippocampus of rats in HIBD group were significantly increased compared with those in sham group. It is suggested that TLR4 pathway is activated after hypoxic‐ischemia, leading to the production of inflammatory cytokines, thus causing inflammatory brain damage. After the intraperitoneal injection of L‐F001, the activation of the TLR4 pathway was inhibited, and the expression levels of related proteins were significantly reduced compared to the HIBD group (Figure 5). It is suggested that L‐F001 may play a neuroprotective role in improving the inflammatory brain damage induced by hypoxic‐ischemia by inhibiting the activation of the TLR4 pathway.

### 3.5 L‐F001 improved growth retardation in HIBD rats

**FIGURE 6:**
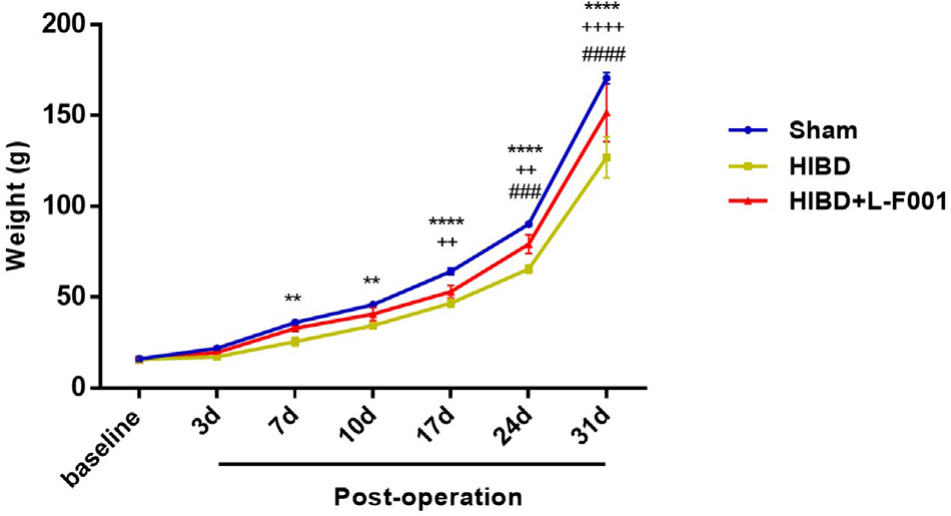
Line chart of body weight changes in each group. Compared to the sham group: ^**^ *p* < .01, ^****^ *p* < .0001. Compared to the hypoxic ischemic brain damage (HIBD) group: ^#^ *p* < .05, ^###^ *p* < .001. Compared to the sham group: ^++^ *p* < .01, ^++++^ *p* < .0001 (*n* = 6).

In order to understand the growth and development of rats in each group, electronic balance was used to measure the body weight of rats in each group before and after modeling. It can be seen from the results that the body weight of rats in sham group increased the fastest, whereas that in HIBD group increased the slowest. The body weight of rats in HIBD group was significantly different from that in sham group at 7 days after modeling, and the difference gradually increased in the later period. After the intraperitoneal injection of L‐F001, the difference in body weight between the treatment group, and the sham group began to appear at day 17. The delay in growth and development improved after treatment. At the same time, there was a difference in body weight between the treatment group and the HIBD group 24 days after modeling, indicating that the body weight of the treatment group was significantly higher than that of the HIBD group (Figure 6). It is suggested that L‐F001 can improve the growth retardation of HIBD rats.

## 4 DISCUSSION

L‐F001 is a good neuroprotective agent with anti‐inflammatory and antioxidant effects (Chen et al., 2017; Luo et al., 2017; Shen et al., 2015). In the neonatal rat model of HIBD, we observed that the hippocampal structure of rats was disordered after hypoxia and ischemia, and the number and volume of Nissil bodies in neurons were reduced. The expressions of pro‐inflammatory cytokines IL‐1β, IL‐6, and TNF‐α were increased and the expressions of anti‐inflammatory cytokines IL‐10 were decreased in plasma of newborn rats. The fluorescence intensity of iNOS and COX‐2 in hippocampus was also significantly enhanced. After the intraperitoneal injection of L‐F001, the hippocampal structure disorder of HIBD rats was improved, the Nissil bodies became deeper, and the expression of the above cytokines was reversed. These results indicate that L‐F001 can improve the inflammatory brain damage caused by hypoxic‐ischemia. At the same time, L‐F001 can inhibit the activation of TLR4/MyD88 pathway in neonatal rat HIBD model, indicating that L‐F001 plays a neuroprotective role after hypoxic‐ischemia by regulating inflammatory response through the TLR4/MyD88 pathway. In the prognosis exploration, we found that L‐F001 significantly increased the body weight of HIBD rats, indicating that it improved the growth and development of HIBD rats. In conclusion, L‐F001 can reduce inflammatory brain damage after hypoxic‐ischemia by inhibiting the TLR4/MyD88 signaling pathway and improve the prognosis of neonatal HIBD rats.

Neonatal HIBD has been widely recognized as a severe inflammatory reaction that ultimately leads to the death of various types of cells in the brain tissue (Li et al., 2020; Wang et al., 2019; Zhao et al., 2020). IL‐1β and TNF‐α are among the best characterized early response cytokines, which have strong pro‐inflammatory effects (Silverstein et al., 1997). IL‐1β level was positively correlated with HIBD severity (Liu & Feng, 2010). Oxidative stress interacts with the inflammatory system and creates a “susceptibility window” for HIBD. There is increasing evidence that TLR4 is a potential therapeutic target for neuroinflammation after hypoxic–ischemic encephalopathy (HIE), and the inhibition of TLR4 has been shown to have a neuroprotective effect on brain damage (Tang et al., 2019). Fasudil, a derivative of 5‐isoquinoline sulfonamide, is one of the most extensively studied ROCK inhibitors. It has the effects of selectively dilating spastic blood vessels, improving cerebral perfusion, reducing inflammatory response, and promoting the regeneration of neuronal axons. It has been shown to have a protective effect on a variety of neurodegenerative diseases (Bowerman et al., 2012). L‐F001, the combination of fasudil and the antioxidant lipoic acid, has been proved in vitro to be superior to fasudil and lipoic acid alone in the anti‐inflammatory and antioxidant effects (Chen et al., 2017; Luo et al., 2017; Peng et al., 2022; Shen et al., 2015). In this study, we further demonstrated in vivo that L‐F001 can reduce the expression of pro‐inflammatory cytokines and improve the structure and function of brain tissue by inhibiting the TLR4 pathway. L‐F001 can not only improve brain degeneration but also improve hypoxic‐ischemia‐induced neurological injury.

Five hours of hypothermia is the standard treatment for newborns at least 33 weeks gestational age at birth with suspected or confirmed hypoxic‐ischemia encephalopathy (Papile et al., 2014). But therapeutic hypothermia does not prevent adverse outcomes in all patients. Randomized controlled trials have found a mortality or disability rate of about 50%. Similarly, although hypothermia treatment can reduce the rate of cerebral palsy and developmental retardation in HIBD neonates, patients with cerebral palsy still account for 19% and those with developmental retardation still account for 23% (Tagin et al., 2012). In the acute stage of HIE, we can improve the outcome of asphyxiated infants by optimizing the stability of the preliminary delivery room. Interventions include limiting the damage of reperfusion and improving supporting hemodynamics and ventilation, thereby reducing the time to recovery of autonomic circulation. Allopurinol, melatonin, n‐acetylcysteine 2‐iminobiotin, remote ischemic posttreatment, cannabinoid, and doxycycline were used in the subacute phase of HIE. Erythropoietin, mesenchymal stem cells, topiramate, and metamedine may be suitable for post‐asphyxia repair (O'Mara & McPherson, 2021; Solevåg et al., 2019; Yıldız et al., 2017). However, the effect of drugs and the applicability of neonates are still not high. The effects and side effects of antioxidant stress drugs currently in research or clinical use are still difficult to predict, but combination therapy is still an important strategy for future neuroprotection of children with HIE. Therefore, the development of neuroprotective agents is a key problem to be solved. In this study, L‐F001 showed good anti‐inflammatory and antioxidant neuroprotective effects in vivo, which could improve the structure and function of hippocampal tissue and ameliorate the growth retardation of HIBD rats. L‐F001 is expected to be a potential therapeutic agent for neonatal HIE.

## 5 CONCLUSION

In conclusion, L‐F001 inhibits TLR4/MyD88 signaling pathway to antagonize neonatal HIBD rats and improve their growth retardation. In the future, we will explore the combined application effect of L‐F001 and hypothermia therapy. More preclinical studies are needed.

## AUTHOR CONTRIBUTIONS

**Ruiyu Zhou**: Investigation; writing—original draft; and formal analysis. **Liqiang Wu**: Investigation and formal analysis. **Sha Sha**: Investigation and writing—review. **Ni Jin**: Writing—review and editing. **Ying Ouyang**: Conceptualization; project administration; and supervision. All authors contributed to the article and approved the submitted version.

## CONFLICT OF INTEREST STATEMENT

The authors declare no potential conflicts of interest.

### PEER REVIEW

The peer review history for this article is available at https://publons.com/publon/10.1002/brb3.3280

## Data Availability

The data that support the findings of this study are available from the corresponding author upon reasonable request.
